# Supplementary material for: Non-invasive longitudinal imaging reveals aging-associated changes in neuroimmune cells in adult zebrafish
Source: bioRxiv. 2025 Sep 25:2025.09.23.676912. Preprint. [Version 1] doi: 10.1101/2025.09.23.676912 (PMC12485939; doi:10.1101/2025.09.23.676912)

bioRxiv preprint doi: https://doi.org/10.1101/2025.09.23.676912; this version posted September 25, 2025. The copyright holder for this preprint (which was not certified by peer review) is the author/funder, who has granted bioRxiv a license to display the preprint in perpetuity. It is made available under aCC-BY 4.0 International license.

Supplemental Figure 1. **A) Fish subjected to longitudinal imaging did not demonstrate weight loss and continued to grow during the imaging period, suggesting that imaging did not interfere with normal feeding behavior or cause excessive stress**. **B)** The time required to resuscitate an individual animal after imaging was not significantly correlated with any measured parameters. Time to Resuscitate vs Age: $p = 0.115$, slope = -1.22, Time to Resuscitate vs Time Imaged: $p = 0.859$, slope = -0.419, Time to Resuscitate vs Weight: $p = 0.071$, slope = 28.82. **C)** Table of tricaine dosage based on stage of anesthesia for fish older than 60 days old (past metamorphic stage).

Supplemental Figure 2. Chamber assembly and full feature diagram. A) The inflow hole and outflow holes (**H.1**) are shown. All holes are tapped using a 1/16-27 NPT standard tap and threading-to-barb fittings (1/16” ID tube) are screwed in. **B)** Holes for screws in the top of the support (H.2) are tapped using a #4-40 UNC tap. Socket screws are screwed in to tighten and loosen the clamp as needed. **C)** Steel dowel pins are placed in H.3, going through the outer chamber and the support piece. A thumb screw is placed in H.4, with only the part of the hole through the support piece being tapped. A M3x0.5 tap is used. The thumb screw must have a 0.5 mm groove starting 3.5 mm from the collar and heading away from the collar. The resulting diameter in the groove must be 3/32”. **D)** Set screws to secure the dowel pins are placed in the side holes (**H.5**). These side holes are tapped with a M2x0.4 tap. **E)** An external retaining ring is placed in the groove on the thumb screw to secure it in place. **F)** A small amount of epoxy is placed in H.6, then magnets are inserted. More epoxy is placed over the magnets to protect them from corrosion. **G)** Magnets are placed in H.7 in the inner chamber using the same method as the previous step. The magnets snap the inner chamber into place but still allow for easy removal. **H)** The digital thermometer probe is placed in the holder (**H.8**) in the inner walls and the blunt end needle and Luer lock adapter is secured in the support (**H.9**).

Supplemental Figure 3. Mounting of fish and imaging setup. A) The restraint device is prepared by wrapping parafilm around the low melting point agarose reservoir. **A’)** The anesthetized fish is placed into the prepared restraint device and tilted upwards (tapping on the benchtop helps fish’s tail slide into the agarose reservoir). Low melting point agarose can be added to reservoir by pipetting and should not fill the reservoir past the dotted line to prevent interference with gills. Caution should be taken to ensure that agarose is an appropriate temperature to avoid burns to fish. **A’’)** A blanket of dental wax can be draped across the fish’s back to further immobilize, with care taken to leave head and gills exposed. Dental wax can be pinched to side of restraint device to secure. Once agarose is set, the device is ready to be

bioRxiv preprint doi: https://doi.org/10.1101/2025.09.23.676912; this version posted September 25, 2025. The copyright holder for this preprint (which was not certified by peer review) is the author/funder, who has granted bioRxiv a license to display the preprint in perpetuity. It is made available under aCC-BY 4.0 International license.

placed into the inner reservoir of imaging chamber. **B)** Example image of fish in restraint device prior to adding dental wax. **C)** Fish placed in restraint device in imaging chamber without addition of dental wax. Intubation tube is placed in the fish's mouth once it is inside chamber. Caution: take care not to position the intubation tube too deeply in the fish's mouth to prevent damage or death to animal. **D)** Schematic of total intubation setup. A perfusion pump is used to circulate warmed system water containing tricaine anesthetic through a pulse dampener and into the imaging chamber. Water is continuously circulated and overflows the top of the inner chamber into the overflow chamber. Water outflows from the overflow chamber and returns to the main carboy where it can be rewarmed and oxygenated.

Supplemental Figure 1

A.

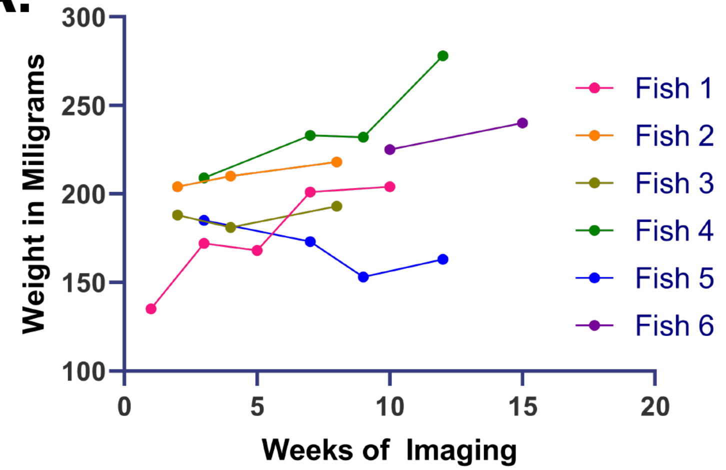

B.

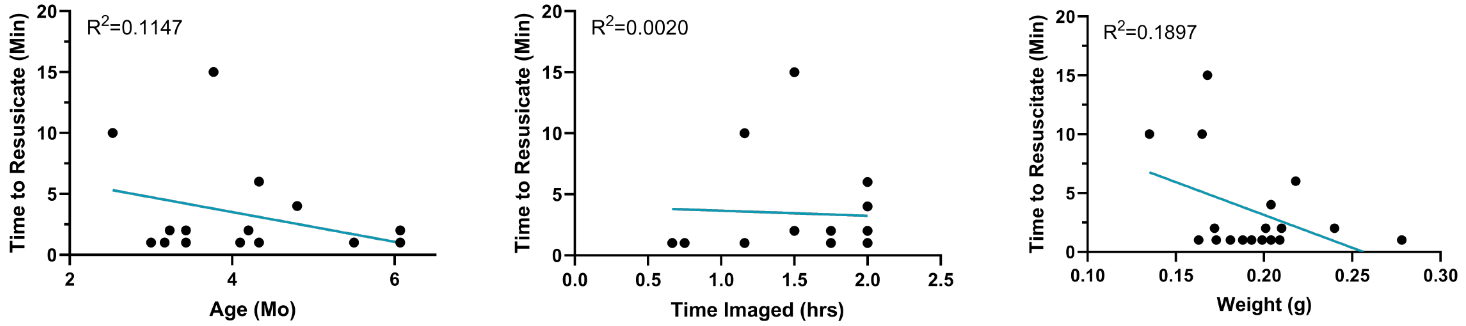

C.

Stage of Anesthesia	Final Concentration (mg/L)	Amount of 10mg/ml Tricaine to add to System Water
**Induction**	180-200	9-10ml to 500ml water
**Maintenance (low)**	150	22.5ml to 1.5L water
**Maintenance (medium)**	160	24ml to 1.5L water
**Maintenance (high)**	170	25.5ml to 1.5L water

Supplemental Figure 2

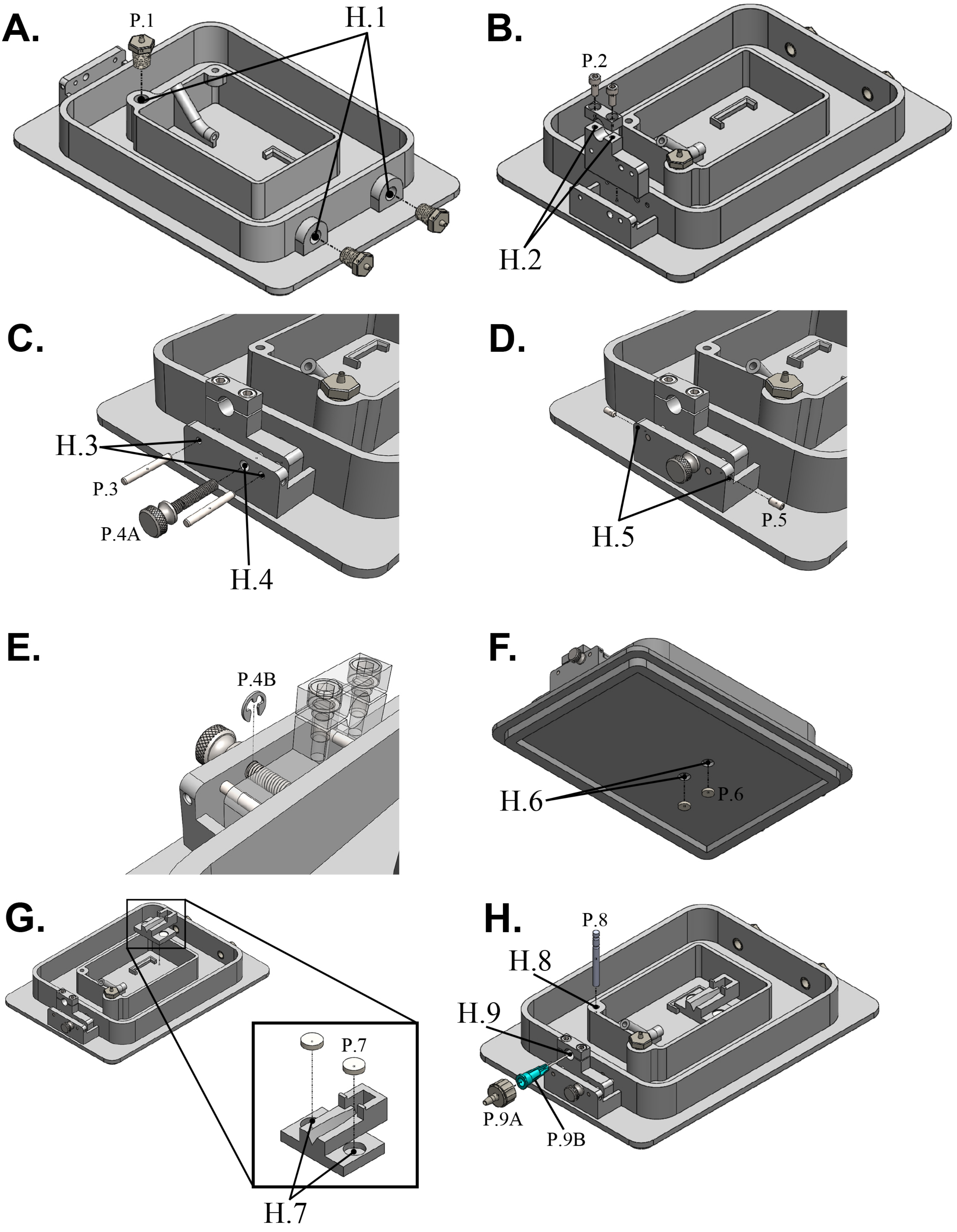

Supplemental Figure 3

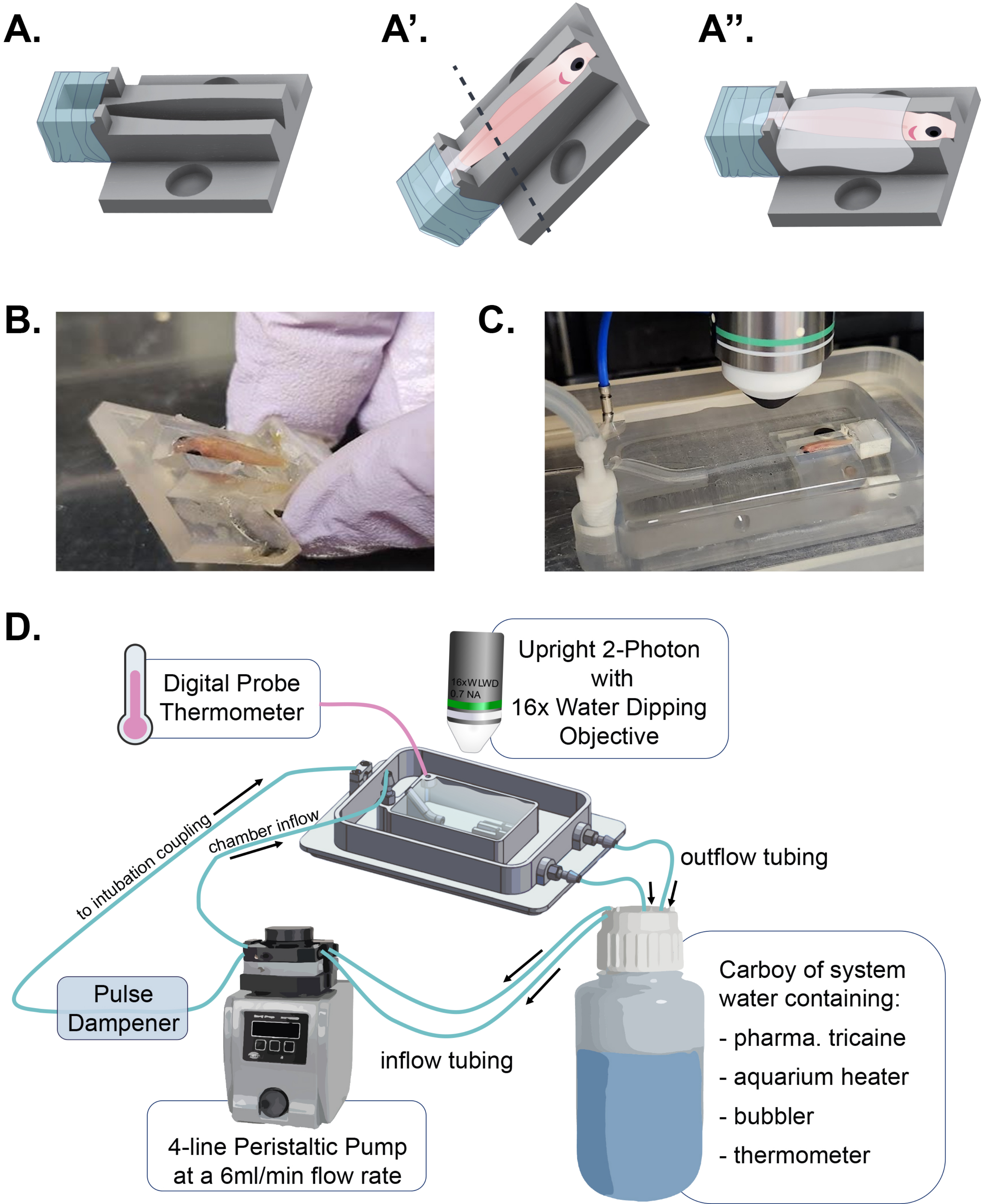

Supplement: Supplementary file 1 [file NIHPP2025.09.23.676912v1-supplement-5.pdf]
